# The Health of Young People in Canada: Focus on Mental Health

**DOI:** 10.24095/hpcdp.45.9.05

**Published:** 2025-09

**Authors:** Wendy Craig, Valerie F. Pagnotta, Stephanie Wadge, Matthew King, William Pickett

**Affiliations:** 1 Department of Psychology, Queen’s University, Kingston, Ontario, Canada; 2 Department of Health Sciences, Brock University, St. Catharines, Ontario, Canada; 3 Department of Public Health Sciences, Queen’s University, Kingston, Ontario, Canada

**Highlights**

- Findings show that mental health is a pressing concern, particularly among cisgender girls and transgender and gender-diverse (TGD) youth. Two-thirds (68%) of TGD youth in Grades 9 and 10 reported feeling sad or hopeless almost daily for two weeks or more, compared to 50% of cisgender girls and 23% of cisgender boys. Only 47% of TGD youth in Grades 9 and 10 reported having a happy home life, compared to 66% of cisgender girls and 80% of cisgender boys. Strong relationships with family, friends and teachers were consistently linked to better mental health outcomes.
- Problematic social media use was reported by 21% of TGD youth in Grades 6 to 8, compared to 7% of cisgender boys and 12% of cisgender girls. Vaping is also a concern, with 29% of cisgender girls and 26% of TGD youth in Grades 9 and 10 reporting lifetime use of vaping products.
- The HBSC Youth Advisory Panel emphasizes the need for improved mental health supports, safe and inclusive schools and greater recognition for the experiences of TGD youth. These youth advisors, aged 11–15 years, provide valuable input to the HBSC reports.
- To access or download the national reports, visit the HBSC web page.

The Public Health Agency of Canada has released *The Health of Young People in Canada: Focus on Mental Health*, a report based on the 2022–2023 Health Behaviour in School-aged Children (HBSC) survey. The study collected data from 26 360 students in Grades 6 through 10 across 317 schools and offers insight into youth health and well-being in Canada. 

